# Wireless and Simultaneous Detections of Multiple Bio-Molecules in a Single Sensor Using Love Wave Biosensor

**DOI:** 10.3390/s141121660

**Published:** 2014-11-17

**Authors:** Haekwan Oh, Chen Fu, Kunnyun Kim, Keekeun Lee

**Affiliations:** 1 Korea Electronics Technology Institute, Seongnam-si, Gyeonggi-do 463-816, Korea; E-Mails: ajounasa@gmail.com (H.O.); kimkn@keti.re.kr (K.K.); 2 Department of Electronics Engineering, Ajou University, Suwon, Gyeonggi-do 443-749, Korea; E-Mail: fuchen92@hotmail.com

**Keywords:** SAW, love wave, biosensor, wireless, batteryfree, IgG, COMP, antenna

## Abstract

A Love wave-based biosensor with a 440 MHz center frequency was developed for the simultaneous detection of two different analytes of Cartilage Oligomeric Matrix Protein (COMP) and rabbit immunoglobulin G (IgG) in a single sensor. The developed biosensor consists of one-port surface acoustic wave (SAW) reflective delay lines on a 41° YX LiNbO_3_ piezoelectric substrate, a poly(methyl methacrylate) (PMMA) waveguide layer, and two different sensitive films. The Love wave biosensor was wirelessly characterized using two antennas and a network analyzer. The binding of the analytes to the sensitive layers induced a large change in the time positions of the original reflection peaks mainly due to the mass loading effect. The assessed time shifts in the reflection peaks were matched well with the predicted values from coupling of mode (COM) modeling. The sensitivities evaluated from the sensitive films were ∼15 deg/μg/mL for the rabbit IgG and ∼1.8 deg/ng/mL for COMP.

## 1. Introduction

In recent years, there has been increasing interest in shear horizontal surface acoustic wave (SH-SAW)-based biosensors for detecting bioanalyte types and concentrations because of their low signal attenuation in liquids, fast response time, high sensitivity, the direct use of raw biological samples for detection, label-free assay, and the simple implementation of array systems. To date, various types of biosensors utilizing SH-SAW have been reported in literature using different materials, designs, and operating principles [1–14]. These SH-SAW biosensors are largely categorized into four general groups depending on their configurations: (1) a two-port delay line biosensor; (2) a one-port reflective delay line biosensor for wireless and battery-free measurements; (3) a resonator-type biosensor; and (4) a Love wave biosensor employing a waveguide layer (overlayer) on the top of the surface acoustic wave (SAW)-delay line. Of these biosensor configurations, the most widely used type is the two-port delay line biosensor, in which one delay line is coated with a sensitive film, while the other is left uncoated for use as a reference delay line [15–17]. The adsorption of a target biomolecule onto the sensitive film changes the velocity of the SH acoustic wave passing through the underneath of the sensitive film using various mechanisms such as the mass loading effect, viscoelastic effect and electro-conductivity effect, resulting in a frequency shift at the output of the SAW device. The frequencies of the two SAW devices are mixed to provide a frequency difference between the two that is directly proportional to the target biomolecule concentration. By using this dual track delay line configuration, SAW Instruments GmbH (SAM5 Green Acoustic Biosensor, Bonn, Germany) released a commercially available, pre-coated, disposable SAW biosensor, which is capable of detecting mass changes on their surfaces that are of the order of several nano-grams per milliliter [18]. Currently, the sensitivity required for the detection of many biological markers on the market is of the order of a few nano-grams per milliliter. However, despite the presence of these commercially available SAW-based biosensors and several other reported SAW-based biosensors, the two-port delay line biosensor has some issues that are yet to be resolved, such as: (I) biological fluids cover the entire delay line surface including interdigital transducers (IDTs), so that a complex test texture should be attached onto the top surface of the biosensor for electrical isolation and further facile biological fluid handling, resulting in unwanted acoustic wave reflections from the test texture; (II) this structural configuration requires complicated measurement systems; (III) there are difficulties with regards to temperature compensation; and (IV) there is a long response time to reach the equilibrium state.

**Figure 1.**
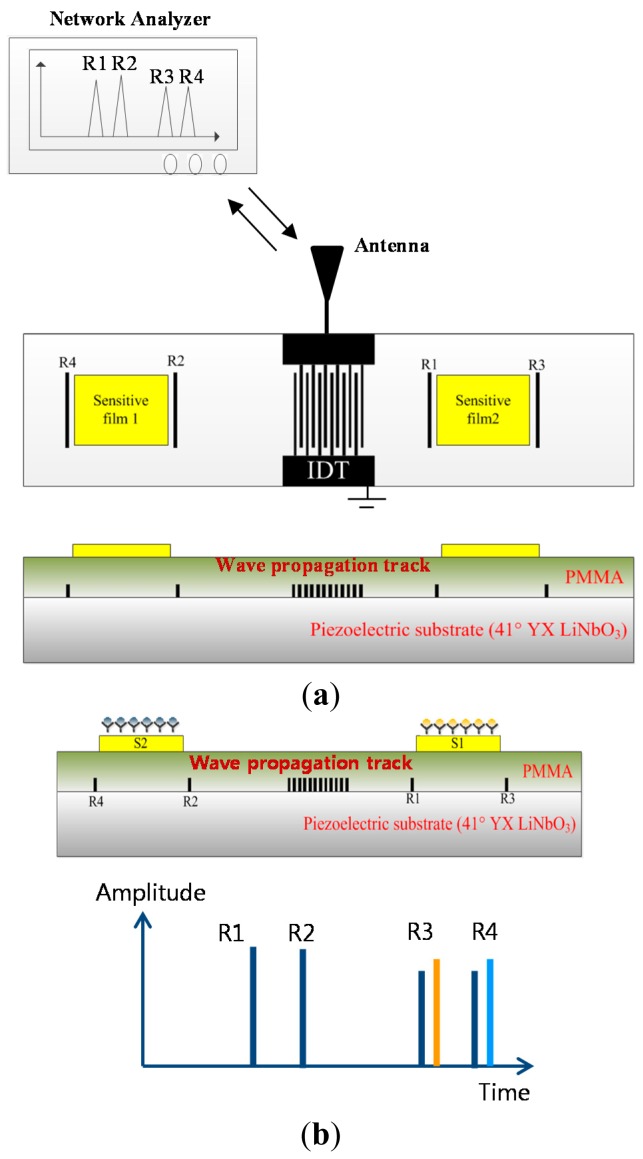
Schematic view of the fabricated device (**a**) from top and cross-sectional view and (**b**) expected reflection peaks derived from four reflectors and two sensitive films in terms of time when analytes are attached onto the sensitive films. Mass loading of bioanalytes onto the sensitive films induces the reflection peak shifts in R_3_ and R_4_.

In this report, we introduce a new type of wireless, battery-free Love wave-based biosensor that utilizes a one-port SAW reflective delay line for the simultaneous measurement of two different biomolecules in a single device. In order to protect IDTs and reflectors from any biofluids and to enhance the sensitivity and stability, we used a Love wave-based biosensor configuration that employs a waveguide layer. Figure 1 shows the entire sensor system, which is composed of a Love wave biosensor, an antenna, and an interrogation unit (network analyzer). When the IDT receives electromagnetic (EM) energy from the interrogation unit (network analyzer) through the antennas, a Love wave is generated and propagates in both directions with evenly distributed power. The propagating Love wave is partially reflected by the reflectors configured with shorted metal stripes and the sensitive films, and the reflected waves to the IDT are reconverted into EM waves at the IDT and transmitted to the measurement system through the antenna. The selective adsorption of target cells to the sensitive films (receptor surface) gives rise to changes in the velocity and amplitude of the Love wave propagating underneath the film because of changes in the density, stiffness, viscosity, permittivity, and elasticity of the sensitive film. The change in the Love wave velocity then results in time shifts of the reflection peaks in the time domain. By evaluating and assessing these shifts, we can extract target cell information. Compared with other currently available wired biosensors, this sensor offers several advantages: no battery installation, absence of a complicated wireless communication circuitry, simultaneous measurement of multiple biocells by a single device, high sensitivity, good protection of the IDT and reflector from liquid environment, and simple temperature compensation.

Coupling of mode (COM) modeling was carried out to determine the optimal device parameters prior to fabrication. Using the extracted optimal design parameters, the device was fabricated and then wirelessly characterized under different antigen concentrations.

## 2. Optimal Design and Coupling of Mode (COM) Modeling

### 2.1. Design Considerations for Simultaneous Detection of Two Different Bio-Targets

The primary goals of this Love wave-based biosensor are to realize a high signal-to-noise ratio, sharp and uniform reflection peaks from all of the reflectors, small spurious peaks, high sensitivity, and stability with temperature variations. To attain these sensor requirements from the one-port Love wave reflective delay line, the center frequency, piezoelectric substrate, IDT and reflector geometries, overlayer material and thickness, and potential binding force between the overlayer and substrate were taken into account to determine the optimized device performance.

A piezoelectric substrate of 41° YX LiNbO_3_ was chosen, because it provides an SH wave, a high SAW propagation velocity (4792 m/s), and a large electromechanical coupling factor *K*^2^ of 17.2% [19–22]. The SH wave with a high *K*^2^ provides a Love wave with a large-amplitude, a high reflectivity from the reflectors, and a low insertion loss. The high SAW velocity facilitates device patterning during fabrication. A one-port reflective delay line configuration is employed for wireless and battery-free measurements, which is composed of a bidirectional IDT and four reflectors on the SH piezoelectric substrate [23,24]. There are two propagation tracks from the bidirectional IDT, as shown in Figure 1. Two reflectors are positioned on the left track of the IDT, and the other two reflectors are placed on the right track of the IDT. Among several possible IDT structures, a bidirectional IDT was used to radiate acoustic waves toward both directions from IDTs with an equal radiation force. The number of IDT pairs determines the bandwidth and the radiation force. An increase in the number of IDT finger pairs will induce a narrower bandwidth and an increased radiation force but will also cause a large static capacitance, which should be removed through impedance-matching circuitry (a parallel connection of a corresponding inductor at the center frequency). Large valued inductor connection for impedance matching at the center frequency can also cause external circuit factors during wide frequency sweeping during the measurement. Therefore, at the design level, we considered an impedance-matched optimal design, which eliminates the need for an external impedance matching circuit at the center frequency.

Based on COM modeling, the number of IDT pairs was set to 25. To reduce the lateral diffraction from the IDT and thus to minimize the insertion loss, the aperture length was set to 80λ. Of the several possible reflector shapes, we chose shorted metal stripes to obtain a higher reflectivity from the reflectors and to reduce any spurious signal between the reflection peaks. To obtain uniform intensities in the reflection peaks, the reflectors close to the IDT have small aperture lengths and few metal stripes, whereas the reflectors far from the IDT have large apertures and many stripes. Generally, an ideal waveguide (overlayer) material should have the following properties: (1) low shear velocity compared to the substrate; (2) high elastic property; (3) low density; (4) low acoustic absorption; (5) good physical/chemical resistance in aqueous or harsh environments, and (6) strong binding force between the waveguide layer and substrate [25–29]. Of the various waveguide material choices such as SiO_2_, polyimide, and benzocyclobutene (BCB), polymethylmethacrylate (PMMA) was chosen as our waveguide material. PMMA has a relatively low density of 1.17 g/cm^3^, a low SH velocity of 1105 m/s and good elastic properties. PMMA also provides a good adhesion binding force with the piezoelectric substrate, as well as a relatively low moisture uptake and swelling. However, during the dicing saw process for the final pieces after completing the device, the PMMA was peeled off from the substrate, resulting in the failure of the device. We therefore require a process that increases the adhesive force between the two layers. To do this, special surface UV/ozone and subsequent chemical surface treatments just before the PMMA deposition was included in the process to boost the binding force between the two layers and to eliminate the peeling affair. Details of this adhesion enhancement process are described in the Experimental Section.

**Figure 2.**
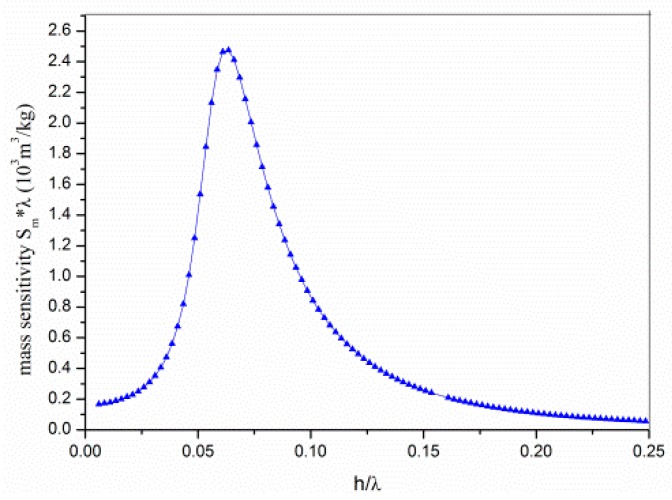
Simulation results for sensitivity *vs.* thickness of overlayer (polymethylmethacrylate (PMMA)). h is the thickness of waveguide and λ is the wavelength at the center frequency of delay line.

There exists an optimal waveguide thickness which provides maximum sensitivity to surface mass loading. For a Love wave device at the optimal waveguide layer thickness, most of the generated Love wave power flows along the upper surface of the waveguide layer, yielding a high sensitivity to any surface perturbation elements. Any further increase above the optimum thickness decreases the sensitivity of the sensor; this is because over-confinement of the wave to the upper surface of the overlayer increases the coupling to the liquid medium. In addition, the insertion loss (acoustic absorption) in the overlayer itself is also increased. If the waveguide thickness is much thinner than the optimum thickness, the waveguide layer does not efficiently trap the acoustic energy near the sensing surface. From the theoretical simulation results, we found an optimal PMMA waveguide thickness of ∼0.6 μm, where the sensitivity of the Love wave sensor was the largest, as shown in Figure 2.

### 2.2. Two Different Bio-Receptor Immobilizations

The sensitive film should have the following properties: (1) high sensitivity to the target; (2) selectivity; (3) fast response time; (4) long-term reliability and stability; and (5) dense binding sites onto the flatform and uniform surface morphology [30–32]. Various methods have been reported in the literature to immobilize receptor molecules on a surface (e.g., physical adsorption, entrapment in a sensitive film lattice, and covalent bonding to the surface). Of all the immobilizing methods, the covalent binding method was considered to be the most promising technique because it allows a reproducible, durable and stable attachment to the film in the aqueous environment. COMP and IgG receptors were chosen as sensitive films. For the covalent bonding of the receptor molecules, first, gold was deposited onto the top surface of the waveguide layer (PMMA). Then, self-assembled monolayer (SAM) treatment was performed to produce monolayers of active groups for the subsequent coupling of the biomolecules onto the surface. Then, a selectively opened polydimethylsiloxane (PDMS) chip assembly (slab) with a high wall was attached onto the waveguide surface for fluid wells. Intermediate layer solutions were dispensed into the fluid wells using the fluid pipette, and then cover-slipped, and then removed from the fluid well, resulting in the immobilization of the receptors on the surface. Finally, a final water rinse was performed to remove the loosely bound receptors from the surface. After completing the process, the PDMS slab can be removed from the surface. Details of these receptor-binding processes are described in the Experimental Section. The total area of both sensitive films was set to 3.5 mm × 1 mm. The reflection peaks from the sensitive films are expected to have a larger amplitude than the shorted grating metal reflectors because of the larger size and mass loading of the film layer itself. Mass loading from the biocell attachment onto the sensitive film changes the velocity of SAW propagating underneath the film, giving rise to a shift in the time position of the reflection peaks. By changing the time delay to phase information, the sensor information was obtained.

### 2.3. COM Modeling

Prior to fabrication, the COM model was used to determine the optimal design parameters. The COM model provides an efficient and highly flexible approach for the modeling of various kinds of SAW devices. The one port reflective delay line was segmented into the IDT and shorted reflector and was then cascaded with a cascading relationship to calculate their total responses. Here, both 3 × 3 mixed matrix (P-matrix) and 2 × 2 mixed matrix representations were used to present the solutions of the COM equations for the IDT and the reflectors, respectively. By solving the P-matrix elements, the admittance matrix (*y*_11_) of the total device and thus the reflection coefficient S_11_ can be obtained by:

(1) $$S_{11}=\frac{\left(Y_{G}-y_{11}\right)}{\left(Y_{G}+y_{11}\right)}$$

where *Y_G_* is the resource and load inductance.

**Figure 3.**
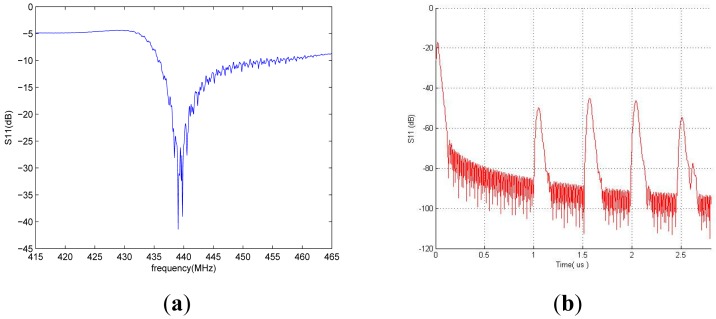
Coupling of mode (COM) modeling results for reflection coefficient S_11_ of one port SAW reflective delay line by using bidirectional IDTs and four shorted grating reflectors in (**a**) frequency domain and (**b**) time domain.

In our simulation, 41° YX LiNbO_3_ was used as the piezoelectric substrate and a 150 nm thick aluminum as IDT and reflector material. To reduce the lateral diffraction from the IDT and thus to minimize the insertion loss, the aperture length was set to 80λ. First, the number of IDT fingers was investigated to determine its impact on the reflection peak. We observed that the optimum IDT should consist of 20 pairs. Moreover, the number of metal stripes for each reflector also plays a crucial role on the sensor performance. Thus, this parameter was simulated. We observed that a larger number of metal stripes can increase the reflector peak but will also induce unwanted multi-reflection signals. As a tradeoff, we suggested the use of five metal stripe fingers for each reflector. Based on these optimum parameters, the total responses of the one port reflective delay line were simulated both in the frequency and time domains, as shown in Figure 3.

## 3. Device Fabrication and Test Setup

### 3.1. One-Port SAW Reflective Delay Line Fabrication

**Figure 4.**
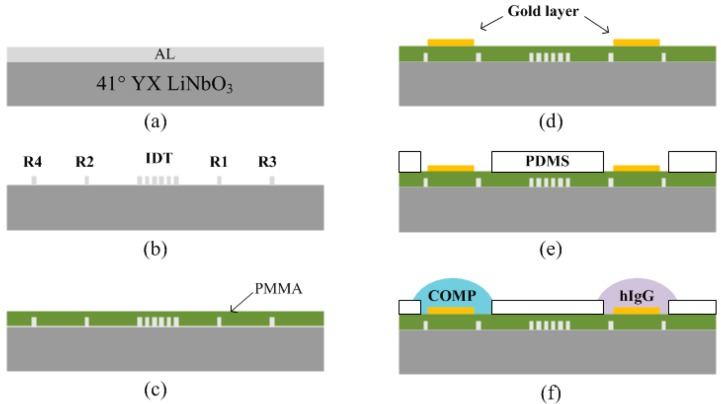
Fabrication procedures. (**a**) Al deposition; (**b**) wet etching of aluminum to complete one port delay line; (**c**) PMMA overlayer (waveguide) spin coating; (**d**) Au deposition and patterning; (**e**) PDMS slab bonding for opened hole; and (**f**) receptor immobilizations onto Au layer.

For sensor fabrication, first, a 41° YX LiNbO_3_ was used as the piezoelectric substrate. A 150 nm thick layer of aluminum was deposited on the 41° YX LiNbO_3_. The photoresist (PR) was spin-coated, exposed, and then patterned for bidirectional IDT and the reflectors (Figure 4). The aluminum was then wet-etched in a solution of 4H_3_PO_4_: 1HNO_3_: 4CH_3_COOH: 1H_2_O. The PR was dissolved in acetone.

### 3.2. PMMA Waveguide Layer

After completing the SH SAW reflective delay line, a layer of PMMA, (obtained from Microchem. Co. Boston, MA, USA) was spin-coated over the entire surface of the LiNbO_3_ substrate. However, during the dicing of the devices to make separate pieces, the PMMA overlayer can be peeled off from the substrate. To enhance the adhesive binding force between the PMMA and the substrate, a special treatment was performed just before PMMA spin-coating. First the samples were treated under UV/Ozone for 10 min. Next, the samples were immersed in a solution of 2.5% (v/v) APTES (3-aminopropyltriethoxysilane) in 95% EtOH for 60 min and then heated at 110 °C for 30 min, a process which enhances the binding force between the two layers. Next, the PMMA overlayer was spin-coated. Different spin rates were used to induce different thicknesses of PMMA on the piezoelectric substrate. The target thickness range was ∼0.6 μm. Then, the PMMA was baked for 30 min at 150 °C to remove all of the solvent for full curing. Next, a 50 nm-thick gold layer was deposited and then patterned on the top of the PMMA surface.

### 3.3. PDMS 3-D Molding and Receptor Immobilizations

There exist two sensitive films over the one-port delay line for the simultaneous measurement of multiple bio-materials by a single device. One is for rabbit immunoglobulin G (IgG) and the other is for COMP (Cartilage oligomeric matrix protein detection [33]. For two sensitive film depositions, a self-assembled monolayer was first formed on the patterned gold surface. Solutions containing 10 mM aminoethane thiol (AET) in water were exposed over the gold sensing areas for 2 h to allow adequate surface adsorption to the polystyrene, and then 1% (v/v) Glutaraldehyde (GA) in pH 8.0 Phosphate buffer with 0.1% NaCNBH_3_ were also dispensed for 1 h. The surface was then rinsed with ethanol and water, and then dried with argon. Next, the PDMS chip assembly was constructed. For the 3D chip assembly, a fully cured PDMS slab was punctured to make holes for two different receptor immobilizations. The selectively punctured PDMS was ozone-treated, attached onto the device, and then pressed to prevent any leakage of the solution during receptor functionalization. The total volume of the opened holes was calibrated referring to the height and areas of the opened holes.

**Figure 5.**
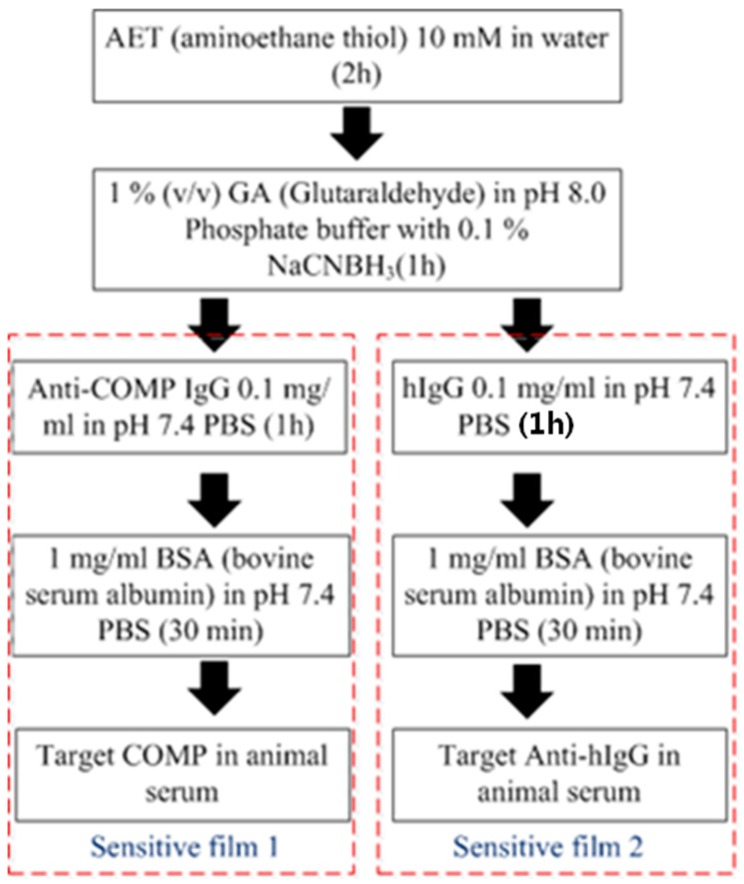
Procedures for two different sensitive film immobilizations.

For COMP receptor immobilization, the surface was exposed to anti-COMP 0.1 mg/mL in pH 7.4 phosphate buffered saline (PBS) for 1 h. The anti-COMP was then reacted with free amine groups. To minimize any evaporation from the receptor fluid, the coverslip was always carefully placed onto the fluid well. After that, the surface was treated with bovine serum albumin (BSA) in pH 7.4 PBS for 30 min to yield the completed antibody sensing layer. Loosely bound anti-COMP from the sensor surface can be removed by a rinsing process or by using high amplitude-acoustic waves during the test. All of the processes were performed at 25 °C (Figure 5). For IgG receptor immobilization, the same procedures were performed. The surface was exposed to anti-IgG 0.1 mg/mL in pH 7.4 phosphate buffered saline (PBS) for 1 h. The anti-IgG was then reacted with free amine groups. After that, the surface was treated with bovine serum albumin (BSA) in pH 7.4 PBS for 30 min to yield the completed anti-IgG sensing layer.

## 4. Results

### 4.1. Completed Devices

**Figure 6.**
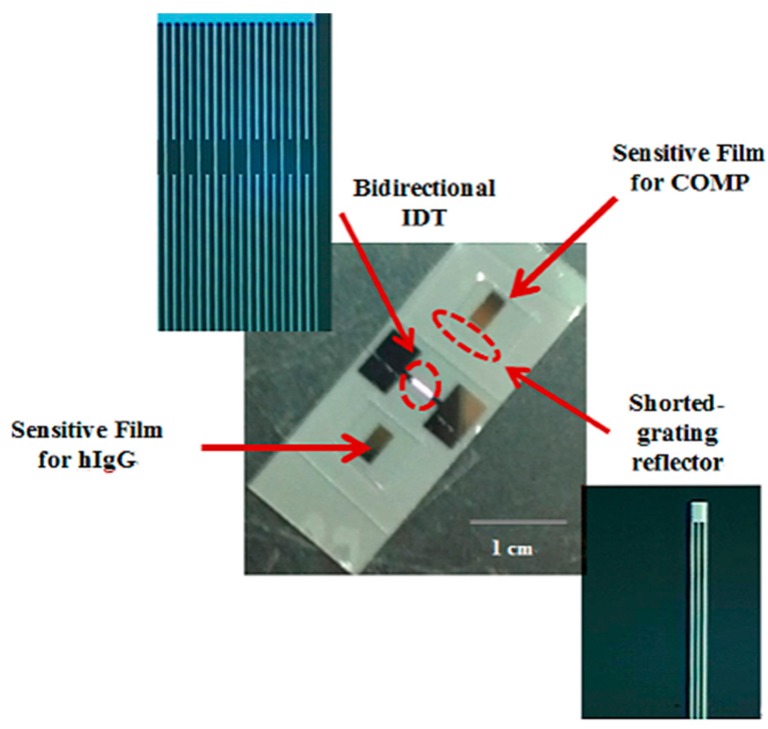
Fabricated one-port Love wave delay line with PMMA overlayer and two sensitive films.

Figure 6 shows the fabricated devices. The IDT and shorted grating reflectors were placed onto the 41° YX LiNbO_3_ substrate for the SAW reflective delay line. A 0.6 μm thick PMMA was formed as the waveguide layer over the reflective delay line. Anti-COMP and anti-IgG receptor layers were selectively functionalized onto the top of the PMMA overlayer for sensitive films.

### 4.2. RF Wireless Testing in Air

**Figure 7.**
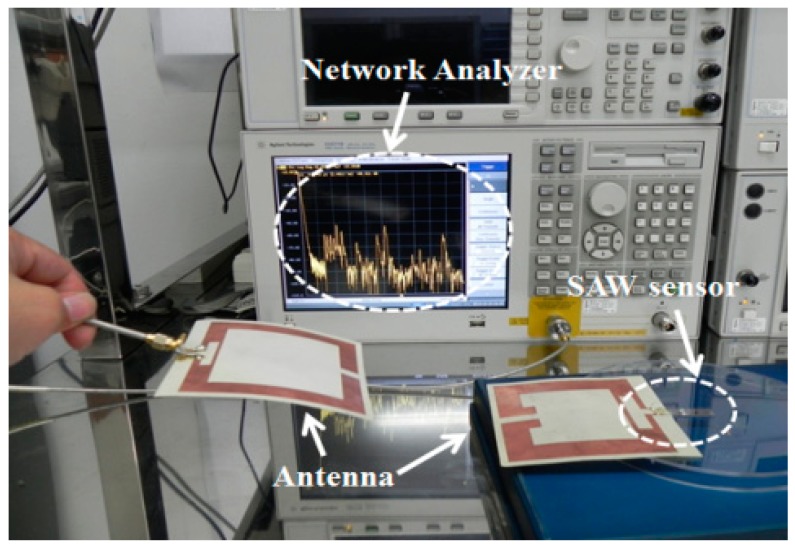
Wireless measurement of the fabricated biosensor with two antennas.

**Figure 8.**
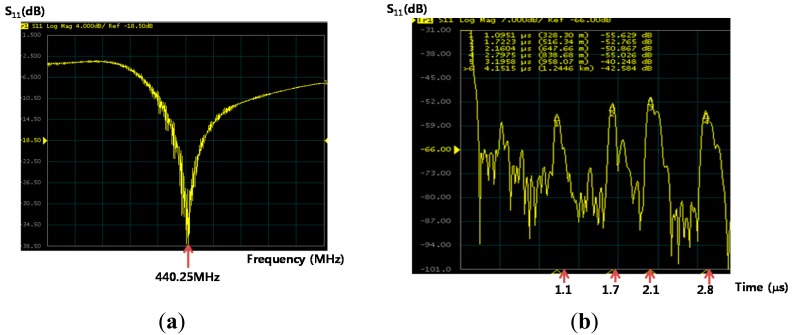
Experimental results of the reflection coefficient S_11_ obtained out of the fabricated device in (**a**) frequency domain and (**b**) time domain. A 440 MHz center frequency and reflection peaks were observed on the network analyzer screen.

**Figure 9.**
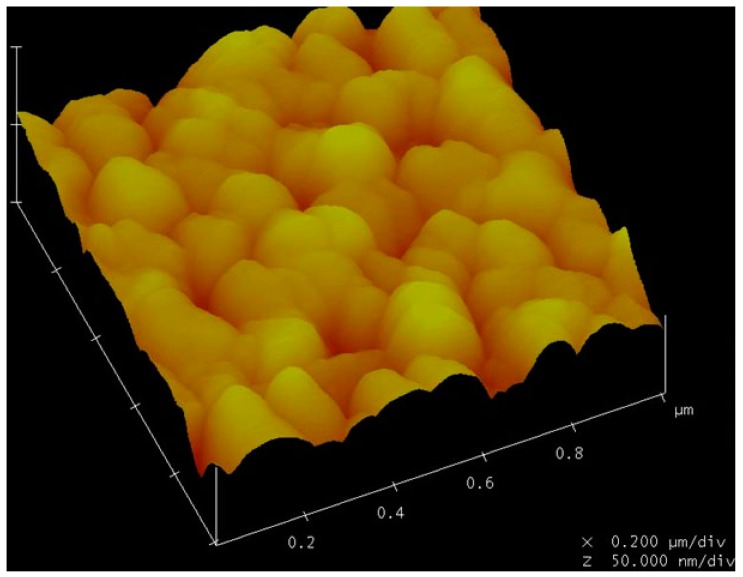
AFM image obtained from rabbit IgG receptor surface. Relatively uniform surface morphology was observed.

The fabricated devices were tested using a network analyzer in air before testing in biosolutions. The two dimensional planar antennas with a 440 MHz operating frequency were fabricated. One antenna was connected to the S_11_ port of the network analyzer and the other was connected to the fabricated biosensor (Figure 7). Using a device with a 0.6 μm PMMA thickness, a 10 dBm RF power was wirelessly applied to the microsensor from the network analyzer. A ∼440 MHz center frequency in the reflective coefficient S_11_ parameter measurement was observed, as shown in Figure 8. These frequency data were converted into the time domain. Sharp, high S/N ratio, uniform reflection peak, few spurious peaks reflection peaks were observed in the time domain. The first reflection peak occurred at 1.1 μs, and at that point, S_11_ was ∼55 dB. The R3 and R4 reflection peaks were obtained from two shorted grating metal reflectors located behind the sensitive films. The obtained experimental peaks were well matched with the simulation data referred in Figure 3. All measurements were performed at 25 °C and with a 20 cm readout distance. Special testing bed was employed to maintain the bed at room temperature (∼25 °C), and the humidity was also set to 40% in absolute. Small temperature variations within the bed did not affect the performances of our fabricated sensor. There were no noticeable performance variations. To evaluate the surface topography of the IgG molecules, AFM measurements were carried out after anti-IgG immobilization. As shown in Figure 9, the AFM image shows evenly distributed rabbit surface profiles, indicating that the anti-IgG receptor layers are well immobilized.

### 4.3. Sensitivity Evaluation under Target Biosolutions

**Figure 10.**
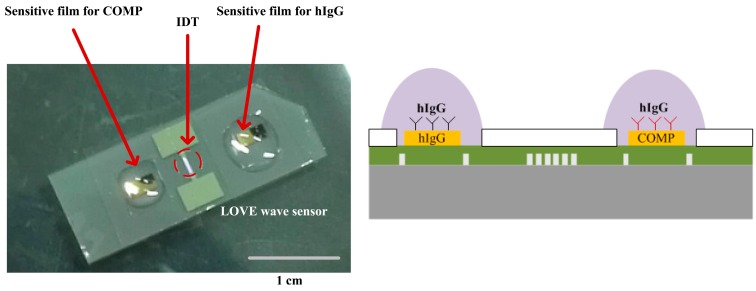
Exact amount of target biosolution was dispensed out of pipette onto opened windows surround by PDMS wall.

**Figure 11.**
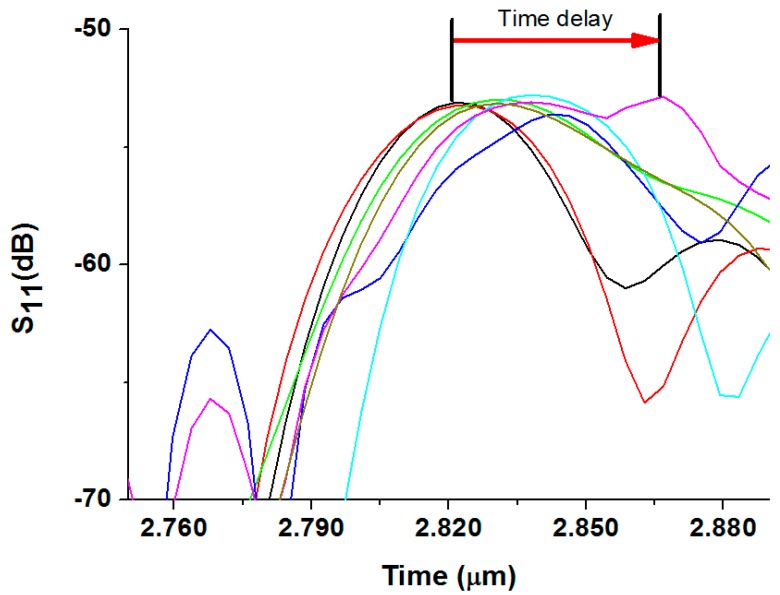
Reflection peak was shifted in the time domain as target biomolecules was selectively attached, and thus the propagating velocity of the acoustic wave was slowed mainly due to mass loading effect.

Accurately measured IgG biosolutions were simultaneously dispensed from a pipette onto the top surface of two sensitive films, and the IgG concentrations were also varied from 1 to 100 μg/mL at each discrete testing (Figure 10). The R3 and R4 reflection peaks were closely analyzed to identify any deviations from its original position. The R4 reflection peak was affected by the rabbit IgG concentrations, whereas the R3 reflection peak was affected by COMP concentration. The position of the R4 reflection peak alone was largely shifted from its original reference position, as shown in Figure 11, because an adsorption of IgG biomolecules onto the sensitive film gives rise to changes in the velocity of the Love wave propagating underneath the film due to changes in mainly the mass loading in the sensitive film. The change in the Love wave velocity induced time shifts of the reflection peaks in the time domain.

**Figure 12.**
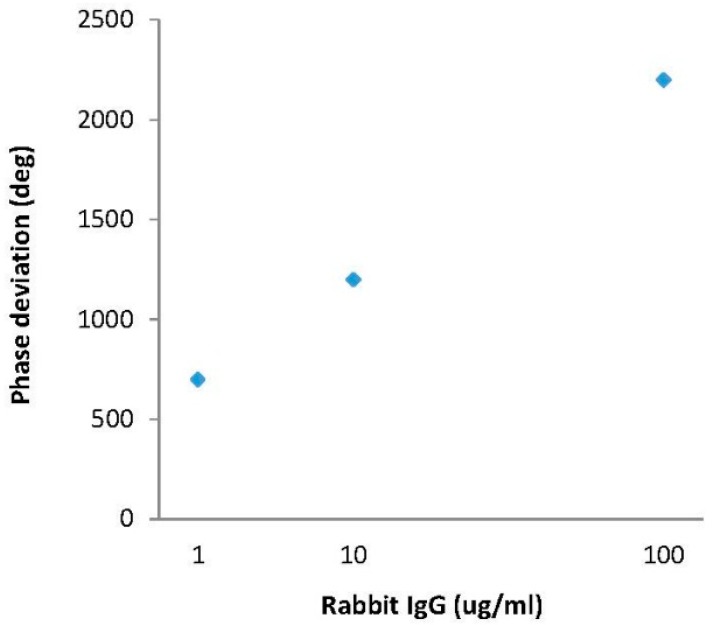
Phase shifts in terms of rabbit IgG concentrations.

**Figure 13.**
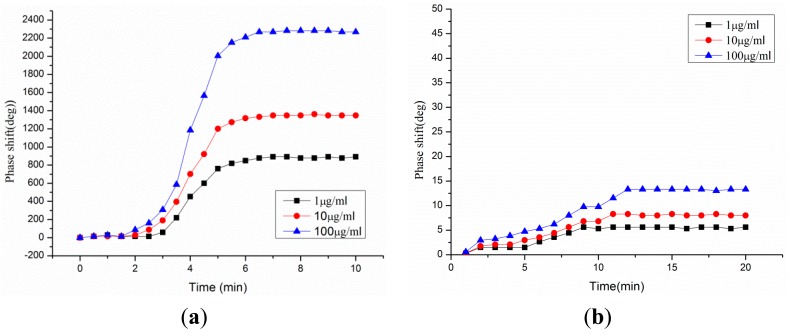
Phase responses of (**a**) the R4 and (**b**) R3 reflection peaks against rabbit IgG concentrations.

The time shifts of the R4 reflection peak were converted into the following phase information (phase = ω Δt) and then were plotted in terms of IgG concentrations (Figure 12). A good linearity was observed in the range of 1–100 μg/mL, and the sensitivity was ∼15 deg/μg/mL for rabbit IgG, which was evaluated from the slope. In contrast, a merely negligible change only was observed at the R3 reflection peak when rabbit IgG solutions were dropped onto the top surface of the COMP receptor layer, because it is related to anti-COMP sensitive film, confirming that our sensitive films have a good selectivity which enables us to discern specific target biomolecules. Next, the response time of the rabbit IgG receptor film was observed in terms of time. Figure 13a shows the phase response of the fabricated biosensor as a function of time for different IgG solutions. The phase response showed a rapid rise upon exposure to rabbit IgG, reaching approximately 80% of the saturation value within 300 s, and was then stable for the remainder of the measurement period. A relatively fast response time (∼5 min), a large phase shift (2200 phase shift for 0.1 mg/mL IgG solution), and high testing stability were observed. However, the phase changes in the R3 reflection peak which is affected by the COMP sensitive film were negligible with changes in the R4 reflection peak, as shown in Figure 13b.

**Figure 14.**
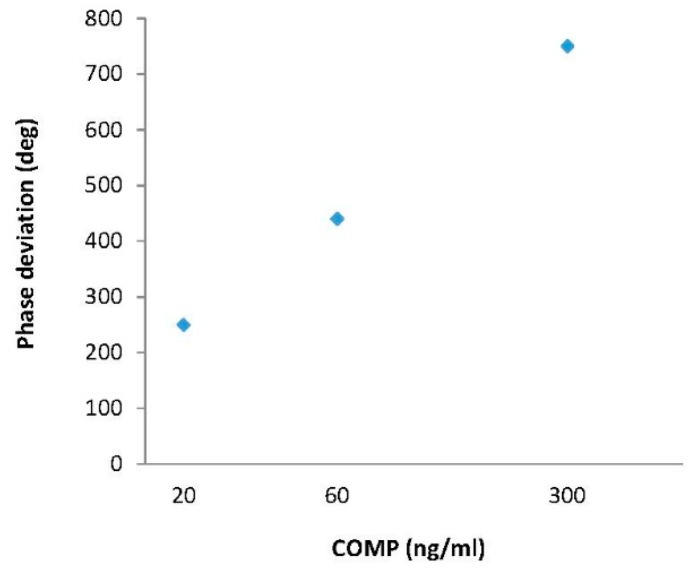
Phase shifts in terms of COMP concentrations.

Using the same method as the IgG testing, the COMP response of the sensor was also characterized under the different COMP concentrations. First, using a pipette, exact amounts of COMP solution ranging from 20 to 300 ng/mL were dropped on both sensing areas. The testing was performed under conditions of 25 °C and ∼20 cm request distance. An attachment of COMP biomolecules on top of the anti-COMP receptor layer induced a mass loading, velocity changes in Love wave velocity, resulting in time (phase) shifts in the R3 reflection peak, whereas the position of the R4 reflection peak which is related with rabbit IgG attachment was merely changed. The time position of the reflection peaks in the R3 reflection peak varied for the different COMP concentrations. The time deviation was converted to a phase angle shift using the following equation (phase = ω Δt). Using the reference point and the R3 reflection peak, we calculated phase shifts. The resultant phase shifts showed nearly linear behavior with increasing COMP concentrations, as shown in Figure 14.

**Figure 15.**
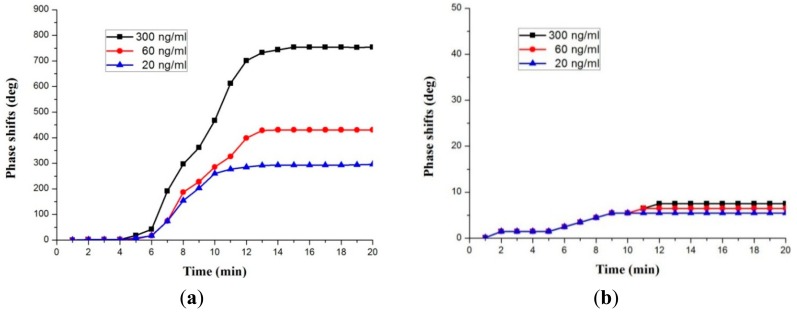
Phase responses of (**a**) the R3 and (**b**) R4 reflection peaks against COMP solution.

The values of the phase shift were 750° for a COMP concentration of 300 ng/mL and 450° for a COMP concentration of 60 ng/mL. It has a linearity within the range of 20∼300 ng/mL in the COMP solution. The evaluated sensitivity was ∼1.8 deg/ng/mL for COMP. From these results, it was clear that the phase shifts increased as the COMP concentrations increased, indicating that this device is sensitive to the binding of COMP to anti-COMP. Figure 15a also shows the phase response of the fabricated biosensor as a function of time for different COMP solutions in the R3 reflection peak. The phase response showed a rise upon exposure to COMP, reaching approximately 80% of the equilibrium (saturation) value within 12 min, which is a somewhat slow response time. However, a large phase shift, and high testing stability were observed. Conversely, only negligible changes were observed in the R4 reflection peak which is related to the rabbit IgG sensitive film as shown in Figure 15b. The negligible phase shifts were observed for long testing periods, confirming that our sensitive films have a good selectivity, enabling the discernment of specific target biomolecules.

## 5. Conclusions

A wireless Love wave-based biosensor was developed for the simultaneous measurement of a variety of concentrations using a single sensor. The Love wave-based sensor was characterized using a network analyzer. Phase shifts were clearly observed when various COMP and rabbit IgG concentrations were dropped onto each sensitive film. Good linearity and sensitivity were also observed for each sensitive film. The sensitivities evaluated from the sensitive films were ∼15 deg/μg/mL for the rabbit IgG and ∼1.8 deg/ng/mL for COMP, demonstrating that the fabricated sensor has a good linearity and selectivity.
